# Editorial: Reviews in the impact of gut microbiota in health and disease

**DOI:** 10.3389/fmicb.2023.1230925

**Published:** 2023-08-11

**Authors:** Junling Shi

**Affiliations:** School of Life Sciences, Northwestern Polytechnical University, Xi'an, China

**Keywords:** gut microbiome, human health and disease, reviews, pathogen, therapy

This topic consists of 15 articles, authored by scholars from 17 different countries, such as China, Canada, Italy, Tunisia, France, the United States, Romania, Spain, Turkey, Pakistan, Croatia, Slovenia, Poland, Brazil, South Korea, Bydgoszcz, and South Africa.

Up to now, the articles have been downloaded 5,241 times and viewed over 23,000 times. The most frequently viewed article is “Human gut microbiota in health and disease: Unveiling the relationship,” which has received 5,423 views.

The published reviews predominantly focus on two aspects: the pathogenic effects of gut microbiota on various diseases, and the application of gut microbiota in therapy. Eleven articles reviewed the correlation between different diseases, including infectious diseases, cerebrovascular diseases, diabetes, Parkinson's disease, and rosacea. These reviews emphasized the pathogenic effects of specific bacteria, such as Helicobacter pylori and Streptococcus. Additionally, an emerging neurometabolic facet of the gut microbiome, known as neuromicrobiology, was highlighted. The methodologies utilized in these reviews included manual summary and analysis, as well as data analysis and visualization using software such as Microsoft Excel 2020, VOSviewer, CiteSpace 5.8.R3, and Co-Occurrence 9.94. Metabolomic analysis was recommended as a powerful tool for exploring the mechanisms underlying the functions of prebiotics and probiotics in the gut health of poultry.

The correlation between gut microbiota and infectious diseases can be explained through the perspective of the human immunological response. The reviews made by Maciel-Fiuza et al. and Afzaal et al., summarized the association between the gut microbial community and the development and progression of various infectious and inflammatory diseases. They also discussed the mechanisms by which disease development is correlated with gut microbiota, specifically focusing on the human immunological response.

Xu et al. summarized the role of the gut microbiome and its metabolites in cerebrovascular diseases. They identified specific gut microbiota and downstream-related metabolites that not only participate in various physiological processes of the human body but also directly or indirectly affect the occurrence and development of cerebrovascular diseases through systemic inflammatory immune response. They further discussed the molecular mechanisms through which gut microbial metabolites regulate the expression of specific interleukins in inflammatory immune responses.

The link between gut microbiota and diabetes has been extensively researched and confirmed globally. Zhang et al., through bibliometrics and visualized studies on publications from 2001 to 2021, found that the understanding of the physiology and pathology of diabetes has been deepened through the lens of gut microbiota.

Papić et al. reviewed the accumulating evidence supporting the identification of microbiota as a potential factor in the earliest, prodromal phases of Parkinson's disease. However, they noted that the link between gut microbiota and neurodegeneration is complex and dependent on various factors. Further research is needed to focus on the metabolic function of gut microbiota in relation to not only motor but also non-motor symptoms of this disease.

Cai et al. reviewed the relationship between gut microbiota and male reproduction. They highlighted how gut microbiota supports male reproduction through nutrition, immunity, and signaling by producing key molecules. They also discussed how gut microbiota helps maintain the integrity of the testes and regulates testicular immunity to protect the spermatogenic environment.

Zhu et al. emphasized the important role of both skin microbiota and intestinal microbiome in rosacea. They indicated *Demodex folliculorum, Staphylococcus epidermidis, Bacillus oleronius, Cutibacterium acnes*, and *Helicobacter pylori* had been identified as pathogens associated with the development of rosacea. Antibiotics and probiotics are commonly used in clinical treatment, and the mechanisms of these treatments were also introduced.

Neuromicrobiology, an emerging aspect of the gut microbiome, highlights the production of neuroactive metabolites by the gut microbiota, particularly neurotransmitters and their precursors. These metabolites stimulate the local nervous system, including the enteric and vagus nerves, which in turn influence brain function and cognition. Miri et al. discussed microbiome-targeted interventions as promising adjunctive treatments using pre-, pro-, post-, and synbiotics. They reviewed the major classes of microbial neuroactive metabolites and emphasized their effects on the microbiome, gut environment, and brain. The authors also discussed the biosynthesis, absorption, and transport of gut microbiota-derived neuroactive metabolites to the brain, as well as their implications in mental disorders.

In addition to the correlation between gut microbiome and diseases, specific pathogens can also play a significant role in the occurrence and development of diseases. *Helicobacter pylori*, as a widely recognized pathogen, has been associated with various gastric diseases, including gastric ulcers, chronic progressive gastritis, and gastric cancer. He et al. elucidated the potential pathogenic role of *H. pylori* in COVID-19, atherosclerosis, hyperemesis gravidarum, and other extragastric diseases. The possible pathogenic mechanisms may involve chronic systemic inflammation and molecular mimicry. Zi et al. summarized the relationship between *Streptococcus* and gastric cancer, as well as the possible carcinogenic mechanisms of *Streptococcus*.

Furthermore, gut microbiota has been explored for its potential therapeutic applications. Four articles discussed the application of gut microbiota in therapy. Fecal microbiota transplantation (FMT) emerged as the most widely used method. The production of butyrate by gut bacteria and the role of *Akkermansia muciniphila* as therapeutic agents were extensively studied. Additionally, “athletic microbiome” is emerging as potential application in therapy.

Wang et al. conducted a bibliometric and visualization study on global research trends and hotspots regarding fecal microbiota transplantation. They identified a total of 57 hotspots related to FMT. Singh et al. suggested that butyrate producers have potential as microbial therapeutics. They explained that these producers generate butyrate from carbohydrates through the butyryl-CoA: acetate CoA-transferase pathway and butyrate kinase terminal enzymes, as well as from amino acids via glutamate and lysine pathways. Butyrate acts as an energy source for colonocytes and maintains an anaerobic environment in the gut. It also helps maintain gut barrier integrity, limit pro-inflammatory cytokines, and inhibit oncogenic pathways. Additionally, colonic butyrate producers shape the gut microbial community by secreting various antimicrobial substances and maintain gut homeostasis by releasing anti-inflammatory molecules.

*Akkermansia muciniphila* is considered a promising “next-generation beneficial microbe.” Li et al. conducted a comprehensive review on this bacterium, which has been extensively studied worldwide since 2004. Clinical uses of *A. muciniphila* have increased over time, and research has been deepened and developed to a more precise level. Oxidative stress has been a prominent focus in related studies.

The concept of the “athletic microbiome” has recently emerged to highlight the potential role of microbiomics in swimmers. As reviewed by Puce et al., training volume/intensity can influence the athlete's microbiome, particularly the non-core or peripheral microbiome, in terms of its architecture, composition, richness, and diversity. Power-/sprint- and endurance-oriented activities, acute and chronic exercise, and anaerobic/aerobic energy systems have differential impacts on the athlete's microbiome. Exploiting microbiomics may have clinical implications, such as assessing the effects of exposure to swimming pools and developing potential pharmacological strategies to address skin infections and inflammation, including acne.

In conclusion, the published articles provided recently reported results on the correlation between gut microbiome and different diseases, as well as the mechanisms and potential application in therapy. The articles may provide useful information for further studies.

## Author contributions

The author confirms being the sole contributor of this work and has approved it for publication.

